# Supplementary material for: Rapid event-related, BOLD fMRI, non-human primates (NHP): choose two out of three
Source: Sci Rep. 2020 May 4;10:7485. doi: 10.1038/s41598-020-64376-8 (PMC7198564; doi:10.1038/s41598-020-64376-8)
Supplement: Supplementary file 1 — Supplementary information. [file 41598_2020_64376_MOESM1_ESM.pdf]

Supplementary Information

Rapid event-related, BOLD fMRI, non-human primates (NHP): choose two out of three

Vassilis Pelekanos, Robert M. Mok, Olivier Joly, Matthew Ainsworth, Diana Kyriazis, Maria G. Kelly, Andrew H. Bell, Nikolaus Kriegeskorte

Supplementary Table S1: Eye-tracking data summary statistics within each session, for all monkeys. The table shows mean and standard deviation of the eye position (degrees of visual angle) across the runs within a session. Although fixations at the fixation cue were not perfectly sustained throughout, the data suggest that eye position variability was reasonable (SD <2° for the majority of sessions) and within the fixation window (which had a size of 5° by 5°).

**Scanning Session**	**Horizontal position, Degrees**		**Vertical position, Degrees**	
**M1**	Mean	Standard Deviation	Mean	Standard Deviation
Session 1	-0.84	1.59	0.89	1.71
Session 2	0.53	1.37	1.21	1.15
Session 3	-1.05	1.46	1.07	1.51
Session 4	0.57	1.18	0.64	1.42
Session 5	-0.18	1.35	1.09	1.40
Session 6	0.57	1.35	1.78	1.13
Session 7	0.00	1.42	-0.33	1.46
Session 8	-0.02	2.01	0.24	1.88
Session 9	-0.67	1.46	0.11	2.04
Session 10	-0.30	1.67	0.81	1.68
Session 11	-0.76	1.68	0.00	1.16
Session 12	-0.64	0.85	0.23	1.05
Session 13	-0.25	1.19	0.86	1.42
Session 14	-1.02	1.34	1.11	1.19
**M2**				
Session 1	-1.00	1.08	2.02	1.31
Session 2	-1.55	1.24	0.90	1.62
Session 3	-0.92	1.09	0.90	1.03
Session 4	-0.40	1.06	-0.69	1.55
Session 5	0.51	1.92	-0.58	2.07
Session 6	-0.52	0.94	1.12	2.05
Session 7	-0.36	1.52	0.46	1.99
Session 8	-0.04	1.75	-0.42	1.93
Session 9	-0.46	1.79	0.25	2.15
**M3**				
Session 1	1.13	1.40	0.86	1.27
Session 2	0.29	1.92	1.67	1.35
Session 3	-1.27	2.26	-0.36	1.65

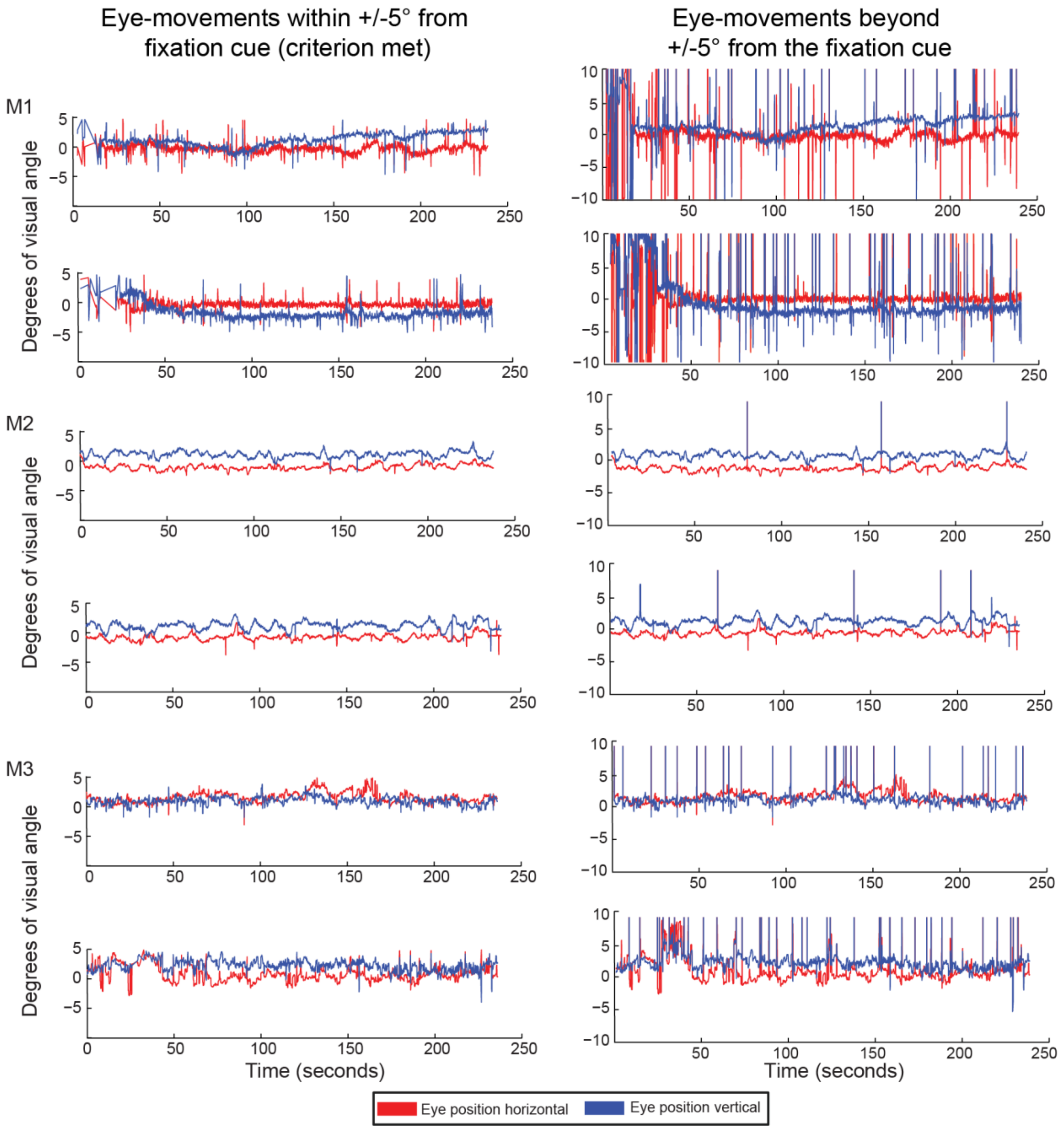

Supplementary Figure S1: Representative subset of the eye-tracking data for all three monkeys. Each graph shows the eye position on the horizontal and vertical plane, expressed in degrees of visual angle, as a function of time. For the purpose of illustrating eye traces in reasonable time chunks, and avoid diffusing the variability between runs due to averaging, each graph shows an individual run. **Left panel:** data that met our fixation criterion. **Right panel:** To highlight which parts of the data were discarded from further analyses, this panel shows, for the same example runs, eye-movement data beyond ±5° from the fixation cue, that is, both the eye-movements that met and the ones that violated our fixation criterion. Summary statistics for the entire list of scanning sessions can be found in supplementary table S1.
